# Decreased expression of TSLP in labial salivary glands of patients with primary Sjögren’s syndrome is associated with local- and systemic disease parameters

**DOI:** 10.1186/1479-5876-10-S3-P36

**Published:** 2012-11-28

**Authors:** Maarten R Hillen, Angela Bikker, Aike A Kruize, Marion Wenting-van Wijk, Floris PJG Lafeber, C Erik Hack, Timothy RDJ Radstake, Joel AG van Roon

**Affiliations:** 1 Rheumatology and Clinical Immunology, UMCUtrecht, Utrecht, the Netherlands

## Background

Thymic Stromal Lymphopoietin (TSLP) is a potent immunomodulatory cytokine involved in Th2-mediated immune responses and homeostatic T-cell expansion. Reduced TSLP expression by intestinal epithelial cells was recently shown to lead to reduced Th2 responses and development of Th1-mediated experimental colitis. Additionally, TSLP is described as a proinflammatory factor in autoimmune diseases. TSLP expression was studied in salivary glands of primary Sjögren’s syndrome (pSS) patients as compared to non-SS Sicca (nSS) patients and the relationship to local- and systemic disease parameters was examined.

## Methods

Tissue sections of minor salivary glands from 38 pSS and 18 nSS patients were stained with a monoclonal antibody (mAb) against human TSLP or an isotype control. In addition, sections were stained with mAbs against CD3, CD19 and epithelial cell marker cytokeratin high molecular weight (CK HMW) or stained with alcian blue to detect mucus production. The number of TSLP-expressing cells was quantified and expression was correlated to local- and systemic disease parameters.

## Results

TSLP was almost exclusively expressed by acinar cells in both pSS and nSS patients. The number of TSLP-expressing cells per mm^2^ was significantly decreased in pSS patients as compared to nSS patients and correlated negatively to Lymphocyte Focus Score (LFS), ESR, serum IgG levels and positively to the percentage of local IgA producing plasma cells. In pSS patients, TSLP was expressed outside of lymphocytic infiltrates at sections that also encompassed high numbers of intact, functional acinar cells as analyzed with CK HMW and Alcian Blue staining.

## Conclusions

TSLP expression is reduced in pSS patients, associated with local- and systemic inflammatory markers.

Considering the described role of TSLP in promoting Th2 responses at mucosal sites, we hypothesize that TSLP is constitutively expressed in salivary glands and promotes a protective Th2 milieu, whereas loss of TSLP expression may contribute to Th1/Th17 associated immunopathology in pSS.

